# The Role of Exosomes in Cancer Progression and Therapy

**DOI:** 10.3390/biology14010027

**Published:** 2025-01-01

**Authors:** Shynggys Sergazy, Roza Seydahmetova, Alexandr Gulyayev, Zarina Shulgau, Mohamad Aljofan

**Affiliations:** 1 LLP VICTUS PHARM, Astana 010000, Kazakhstan; shynggys.sergazy@gmail.com (S.S.); rozabat@mail.ru (R.S.); agulyayev@nu.edu.kz (A.G.); zarina.shulgau@nu.edu.kz (Z.S.); 2 National Laboratory Astana, Center for Life Sciences, Nazarbayev University, Astana 010000, Kazakhstan; 3 Department of Biomedical Sciences, School of Medicine, Nazarbayev University, Astana 010000, Kazakhstan

**Keywords:** exosomes, drug delivery, tumor-derived exosomes, cancer therapy, extracellular vesicles, tumor microenvironment

## Abstract

Exosomes are small extracellular vesicles and are crucial in intercellular communication. Interestingly, tumor-derived exosomes carry oncogenic molecules, such as proteins and microRNAs, which can reprogram recipient cells, promote angiogenesis, and stimulate cancer pre-metastatic niche, supporting cancer growth and metastasis. On the other hand, their biocompatibility, stability, and ability to cross biological barriers make them attractive candidates for drug delivery. Recent advances have shown the potential for exosomes to be used in early disease detection and in targeted drug therapy by delivering therapeutic agents specifically to tumor sites. Despite the promising applications, a number of challenges remain, including exosome isolation and characterization, as well as their inherent heterogeneity. Thus, the current review aims to describe the roles of exosomes in health and disease, and discuss the challenges that hinder their development into becoming useful medical tools.

## Simple Summary

Exosomes are small particles released by cells and involved in cellular communication. In cancer, they carry signals from tumor cells to other cells, encouraging cancer to grow and spread. This review describes how exosomes play two opposing roles at the same time, consisting of how they contribute to cancer progression and how they might be used to treat cancer in new ways. On the one hand, tumor-derived exosomes carry certain molecules that alter adjacent cells to support the tumor, help cancerous cells move to other parts of the body, and avoid the defense of the immune system. On the other hand, exosomes could be used as a suitable means to deliver cancer-fighting drugs directly to tumor cells, making treatment more targeted and potentially reducing side effects. However, a number of challenges could slow down their development into useful medical tools. Thus, this work aims to describe exosomes, their components, and the current challenges for their future use.

## 1. Introduction

Exosomes are small lipid bilayer particles (measuring 30–160 nm) secreted by various cells, and have been shown to be involved in cellular communication, contributing to various aspects of cell physiology and pathophysiology. More than four decades ago, they were described as cell-secreted particles carrying membrane-bound enzymes taken up by recipient cells, making them a potential means of cellular communication [1]. Their particle diameter allows them to act as nano-carriers, facilitating deep penetration into tissues and overcoming barriers such as the blood–brain barrier and deformable cytoskeleton [2]. Their nanoscale allows them to transport cargos of proteins, miRNA, mRNA, DNA, and lipids.

Exosome biogenesis is a highly regulated and complex process that involves different consecutive stages, that begins with endocytosis of the particles followed by cellular budding, invagination, multivesicular body (MVB) formation, and ends with secretion of fully mature exosomes into the extracellular environment [3]. The membranes folding creates intraluminal vesicles (ILV) that include the majority of the cytosolic components, such as proteins, lipids, and nucleic acids [4]. Up until the last decade, exosomes were thought to primarily serve as carriers for cellular waste, but increasing research indicates that they play a crucial role in intercellular communication [5]. However, the selective sorting of the different molecules into the budding exosome vesicles has appeared as a crucial factor in exosome functionality, diversity, and specificity [6].

Exosomes, being the smallest member of the extracellular vesicles, which also include microvesicles (50–1000 nm) and apoptotic bodies (500–2000 nm), have emerged as a promising alternative to nanoparticles for therapy and drug delivery purposes [1,2]. Their nano-scale size range, enhanced biocompatibility, excellent cargo capacity, reduced immunogenicity compared to polymer-based alternatives, passive targeting and enhanced permeability and retention effects make them suitable carriers for drug delivery [7]. They also exhibit high biological stability, even for long-distance intercellular communication [8], and can deliver their cargo into the intracellular environment of the organism, due to specific molecular compositions on their surface, which enable fusion with cell membranes [9,10,11].

However, exosomes can encapsulate a wide array of bioactive molecules, including immunomodulatory chemicals, such as cytokines, chemokines, and cytotoxic proteins, that can affect the activity of immune cells [12]. Immune cells-derived exosomes, such as those from dendritic cells, macrophages, and T cells, play a crucial role in immunological regulation in both health and disease. For example, exosomes derived from dendritic cells can affect other types of immune cells, to either inhibit or stimulate an immune response [13].

Nonetheless, exosomes have the potential to contribute to the treatment of cancer as well as other diseases. While they may have a wide range of potential, clinical uses, including as a drug delivery system, and biomarkers for early detection and treatment of disease, they have also been reported to promote cancer progression and might stimulate cancer metastasis. In addition, a number of challenges remain to be addressed in order to fully understand the potential of exosomes in clinical settings, including their isolation, characterization, and the implications of their use as a friend or a foe. Also, other challenges that might limit their utilization include loading efficiency and their production by recipient cells, as well as their ability to target tumor cells [14]. Thus, the current review aims to describe exosomes, their components, and their signaling pathways, in both health and disease, as well as the challenges facing their clinical utilization.

## 2. Exosomes in the Tumor Microenvironment

The tumor microenvironment is a complex and dynamic signaling network that involves various types and functionalities to modulate enormous cell-to-cell communication, which controls different stages of cancer initiation and progression [15]. The tumor environment, which is a product of sophisticated crosstalk between different cells, is thought to be of high importance in cancer diagnosis and treatment [16]. The crosstalk presented by cellular signaling and communication networks in the tumor environment plays important roles in cancer development, progression as well as treatment [17,18,19]. This tumor ecosystem not only contains cancer cells, but non-cancer cells such as endothelial cells, cancer-associated fibroblasts, and immune cells, which have different compositions and functions depending on various factors including patient characteristics, the tumor type and stage, and the organ where the tumor originated from [20]. The cancer cells in this complex ecosystem use dynamic processes to remodel the vasculature and extracellular matrix, as well as reprograming non-cancerous cells to generate a heterogenic tumor-supportive environment [21].

Interestingly, the heterogeneity of the tumor microenvironment, which reportedly exists in all cancer types, has been implicated as one of the main sources of resistance against anticancer treatment, as well as a major contributor to lethal outcomes [22]. Thus, measurement of tumor heterogeneity might be a useful prognostic marker [23]. This environment is maintained by cellular signaling, which is a complex process involving various interacting molecules that operate in the context of networks, some of which could play a synergic role; others might induce an antagonistic effect [24]. Some of the plethora of molecules involved in these complex and interconnected synergistic and antagonistic networks include secreted factors, such as chemokines, cytokines, and extracellular vesicles including exosomes, which show the complexity and dynamic nature of cellular signaling in the tumor microenvironment [24]. Furthermore, tumor tissues have been found to contain significantly more exosomes than normal tissues, indicating the frequency and complexity of intercommunication between tumor cells, and depending on their cargo, exosomes can either suppress or promote tumor progression and proliferation [25].

## 3. Tumor-Derived Exosomes Can Initiate Tumor Growth

Exosomes appear to play various and conflicting roles in tumor initiation, promotion, and progression. For example Skog et al., in their study of the transport of RNA and tumor promoting proteins from glioblastoma-derived exosomes, claimed that glioblastoma-derived exosomes containing mRNA, microRNA (miRNA) and angiogenic proteins promote angiogenesis when taken up by normal host cells, such as brain microvascular endothelial cells [26]. Their study reported that tumor-derived exosomes contain angiogenic proteins and stimulate tubule formation by endothelial cells as well as causing proliferation of a human glioma cell line, suggesting a self-promoting feature.

Exosomes regulate extracellular communication and are considered critical mediators in the progression of cancer; they are, reportedly, capable of transforming fibroblasts into cancer-associated fibroblasts [27,28]. For example, exosomes derived from colorectal cancer increased the expression of α-SMA, a marker for cancer-associated-fibroblasts [29]. In addition, prostate cancer-secreted exosomes contain TGF-β and might possibly activate fibroblasts [27]. Exosomes derived from cancer cells increase angiogenesis in cancer progression [24]. This might likely be due to the abundance of angiogenesis-related factors in exosomes, such as miRNAs, mRNAs, and angiogenic proteins [30]. This might cause phenotypical and functional changes in stromal cells, which lead to an increase in the cells’ ability to proliferate and migrate [30].

Interestingly, several biological processes are regulated by miRNA, including those that secrete from cells via the exosome, which are involved in cellular signaling in various conditions (physiological and pathological) via controlling different target genes [8]. Furthermore, research has showed that exosomal miRNAs play an essential role in facilitating cell–cell communication, particularly between endothelial cells, cancer and immune cells [12,13,15,17,18]. While their role is still not fully understood, tumor-derived exosomes were shown to modify the microenvironment of adjacent cells and initiate metastatic mechanisms [31]. In their study that investigated the effect of exosomal transfer of angiogenic on cancer metastasis, Kosaka et al. revealed that transfer of exosomal mRNA, particularly, miR-210 from metastatic cancer cells, controls the microenvironment of endothelial cells in favor of the cancer cells in a way that stimulates cancer metastasis. Their study claimed that preventing the expression of neutral sphingomyelinase 2 in metastatic cancer cells inhibits the metastatic capabilities of tumor cells in targeting lung tissues, and that their re-formation through the administration of metastatic-derived exosomes reverses the effect [32]. Similarly, Li et al., who investigated the role of exosomal mediated secretion of lysyl oxidase-like 4 on the ability of hepatocellular carcinoma cell invasion and metastasis, reported that hepatocellular carcinoma-derived exosomes with lysyl oxidase-like 4 caused tumor invasion and metastasis in human umbilical vein endothelial cells (HUVECs) [33]. This could suggest that the secreted lysyl oxidase-like 4-containing exosomes may serve as stimuli, in either autocrine or paracrine fashion, to stimulate metastasis of hepatocellular carcinoma cell via tumor–stromal cell interactions, further proving the potential effect of cancer-derived exosomes as metastasis promoters. While the mechanism is unknown, it was reported that, under hypoxic conditions, endothelial cell-derived exosomes increase extracellular matrix crosslinking by upregulating lysyl oxidase-like 2, thus impacting the tumor microenvironment [34]. Nevertheless, exosomes seem to participate in tumor development not only through molecular pathways, but also through genetic mechanisms. They have been implicated in the stimulation of tumor proliferation and progression via alteration of gene expression [35]. For example, Sun et al., 2020 described the role of ubiquitination and deubiquitination in cancer metabolism, reporting that exosomes are frequently involved in post-translational modifications including ubiquitination [36]. However, dysregulation of ubiquitination and deubiquitination, which form an integral regulatory part of tumor metabolic reprogramming have been reported as potential contributors to various diseases, including cancer [25,37].

## 4. Molecular Cargo of Tumor-Derived Exosomes

**Figure 1:**
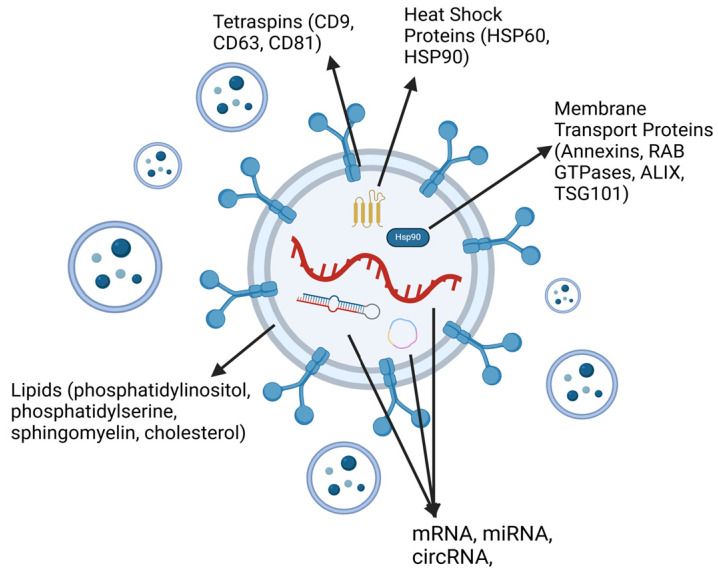
Molecular cargo of tumor-derived exosomes.

Exosomes contain a cluster of heterogeneous particles, reflecting the cell type from which they were secreted (Figure 1). The molecular cargo of exosomes seems to depend on the cell type as well as the origin of the cells. Garnier et al., who investigated qualitative changes in the proteome of extracellular vesicles associated with cancer cell transition to mesenchymal state, reported that the exosomes secreted from human squamous cell carcinoma undergoing EMT-like stimulation also displayed a similar proteome reprogramming [38]. This is further supported by Wen Wen et al., who claimed using proteomic analyses that exosomes derived from breast cancer reflect the phenotype of the parent cell; for example, secretome of exosomes mirrors the epithelial/mesenchymal phenotype of the secreting cancer cells [39]. The molecular cargo of tumor-derived exosomes appears to differ significantly from those produced by normal cells, and, in fact, tumor cells were reported to produce significantly higher numbers of exosomes compared to normal cells [40]. Exosomal functions appear to depend on their protein content, with proteomic analysis of exosomes from patients with various malignant tumors showing that exosomal protein levels in the plasma are associated with the tumor grade, stage, activity, therapeutic response, and survival [41].

Exosomes contain various types of proteins that are mostly associated with membrane transport, including annexins, RAB GTPases, ALIX, the MVB-producing protein, and flotillins, as well as 101 protein (TSG101), which is a tumor susceptibility gene [42]. In addition, they express tetraspanins proteins, which are small transmembrane proteins present on the cell surface of almost every eukaryotic cell, including the heat shock proteins, HSP60 and HSP90, as well as CD9, CD63, and CD81 [43,44]. They also contain lipids such as phosphatidylinositol, (PI), phosphatidylserine (PS), phosphatidic acid (PA), sphingomyelin (SM), and cholesterol [45]. Another important component contained within exosomes is nucleic acids that modulate gene expression and may potentially be used as biomarkers [46]. A number of nucleic acids are reported to be found in exosomes, including, mRNA, microRNA (miRNA), snoRNA, and circRNA, lncRNA, and tRNA [47,48].

## 5. Cellular Communication and the Role of Exosomal Molecular Cargo

Cancer cells have developed complex mechanisms to suppress cellular immunity, including the release of tumor suppressing factors that inhibit T-lymphocytes and of dendritic cells development. Tumor cells display and use a number of immunosuppressive molecules to hinder immune responses [49]. Immuno-evasion is one of the crucial steps that cancer cells undertake to survive and proliferate. Thus, a potently prolonged immune response against cancer cells and a reduction in immunosuppression are crucial for effective anticancer treatment. Generally, exosomes carry various types of molecular cargos and their composition is different depending on the type and health of the secreting cells. Exosomes carry parts of their molecular signature from their parent cells, which differentiates between exosomes produced by different cells, and is quite different between tumor-derived exosomes and healthy cell-derived exosomes [50]. Cancer-derived exosomes contain various immune-suppressing chemicals, including transforming growth factor beta (TGF-β), a crucial cytokine for cellular proliferation, differentiation and morphogenesis [51,52]. While TGF-β has various functions in the tumor microenvironment, it was also reported to facilitate the formation of CAF, which results in the increase of extracellular matrix production, leading to the establishment of an immunosuppressive environment [53].

Another reported immunosuppressive mechanism of TGF-β-containing exosomes is when TGF-β joins interleukin-2 (IL-2) to induce the transcription factor Scurfin (FoxP3), which controls T-dependent immune responses by regulating of CD4 + T cell [54]. Along with IL-2, TGF-β facilitates the activation of SMAD proteins, a family of inducible transcription factors that recruits NFAT, which are key regulators of T-cell development and function to promote the gene FOXP3, inducing the expression of FOXP3 mRNA [55,56]. Furthermore, tumor-derived exosomes expand and increase, in a dose-dependent manner, the immunosuppressive function and population of T-regulatory cells, particularly CD4 + CD25 + FOXP3+ [57]. Cells that were co-cultured with tumor-derived exosomes showed an increase in the expression of levels of cytotoxins and immunosuppressive cytokines, including CTLA-4, TGF-β1, FasL, perforin, granzyme B, and IL-10 [57]. Exosomes derived from patients with malignancy significantly hindered further reduction in the numbers of Treg and the expression levels of FOXP3 in a proportional manner to that of TGF-β1 [58]. Other reported proteins that were expressed in tumor-derived exosomes including TRAIL, a death receptor ligand; in addition to inhibitory cytokine TGF-β1, they also contain IL10, prostaglandin E2 (PGE2), and PD-L1, which is a check point receptor ligand [50,59].

## 6. Exosomes and Cancer Therapy

### 6.1. Exosomes as a Drug Delivery System

Several characteristics of exosomes make them attractive candidates for targeted drug delivery. Their role in cellular communication in both adjacent and remote cells, and their ability to carry various components intracellularly, make them an ideal candidate in, for instance, delivering anticancer drugs, in that they can reduce toxicity and lower immunogenicity in targeted delivery [60]. Importantly, exosomes can efficiently cross biological barriers, such as the blood–brain barrier, mucosal barriers, and the placental barrier [61]. They were also reported to be amenable to molecular modification, thus providing a possible means to enhance their therapeutic efficiency [62,63]. For instance, exosome-coated drugs, such as adriamycin, paclitaxel, and sorafenib, have demonstrated the potential to reduce drug side effects and enhance treatment efficacy [64]. Another advantage is that exosomes are available in abundance and that they can be sourced from a broad range of sources, including animal-origin exosomes and plant-derived exosome-like nanoparticles. Regardless, of the source, exosomes exhibit diverse biological characteristics and offer clinical advantages in various cancer treatment approaches [65,66]. Recently, we investigated the suitability of mare’s milk-derived exosomes as a potential drug carrier for the naturally occurring polyphenol, quercetin [67]. The in vitro results showed no difference in antioxidant activities between 160-µM of free quercetin and 80-µM quercetin loaded into mare’s milk-derived exosomes. Furthermore, quercetin loaded into exosomes, but not free quercetin, was able to reverse acute doxorubicin-induced oxidative stress in rat models [67]. This indicates that exosomes were able to improve the bioavailability of quercetin, thus confirming their suitability as a potential drug delivery system.

### 6.2. Exosomes as Biomarkers for Early Disease Detection and Monitoring

The stability of the exosomal membrane structures and the widespread availability of exosomes in bodily fluids make them promising biomarkers. A promising cancer diagnostic tool that utilizes exosomes is exosome-based liquid biopsy, which is anticipated to become a valuable tool in future in the early diagnosis of cancer [64,68,69]. A summary of some of the reported functions is presented in Table 1. Exosomes possess characteristics that allow them to be utilized as early disease biomarkers for the diagnosis and treatment of cancer. For instance, exosomal miRNAs, such as miR-25-3p and miR-21-5p, have been reported to promote vascular permeability and angiogenesis, leading to cancer metastasis [70,71]. Li et al., 2018 reported that gastric cancer-derived exosomal miR-21-5p promotes peritoneal metastasis through the mesothelial-to-mesenchymal transition [72]. This finding is further supported by He et al., 2021, who reported that exosomal miR-21-5p extracted from cancer cells induced cellular angiogenesis and vascular permeability [71]. However, the latter study claimed that this occurs through suppressing of the Krev interaction trapped protein 1 (KRIT1), a scaffolding protein that promotes endothelial adherens junction stability in recipient cells, leading to the activation of the β-catenin signaling pathway, thus increasing vascular endothelial growth factor and cyclin D1, and resulting in angiogenesis and vascular permeability [71].

**Figure 2:**
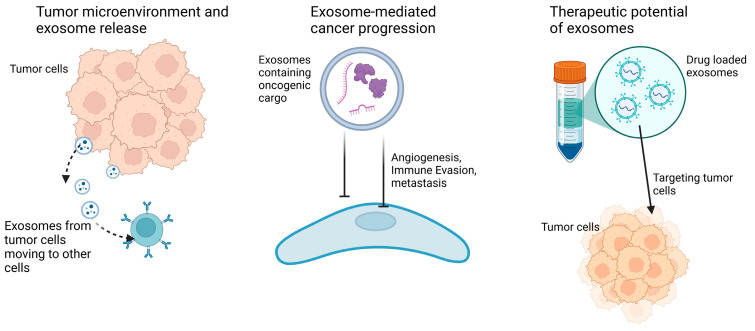
Exosomal roles in cancer growth and progression.

Furthermore, transfer of cancer-derived exosomal miR-21-5p from cancer cells to endothelial cells stimulates angiogenesis and vascular permeability [73]. Similarly, elevated expression levels of miR-3157-3p in circulating exosomes have been observed in patients with metastatic non-small cell lung cancer, compared to patients with the non-metastatic form [74]. The critical role that exosomal miR-3157-3p plays in pre-metastatic niche formation makes it a possible blood-based biomarker for cancer metastasis. These findings highlight the potential of use of exosomes as biomarkers for disease monitoring and prevention, reflecting the metabolic state of their originating cells and their role in key pathogenic processes. Consequently, precise identification and disease-specific characterization of exosomes are essential for their diverse applications in disease research and diagnostic procedures [75]. Figure 2 summarizes some of the reported roles of exosomes in cancer progression and therapy.

**Table 1:** Extracellular Vesicles (EVs) in Cancer Progression and Therapy.

Role	Mechanism of Action	Biomolecular Pathways	References
Promotes Angiogenesis	Transfer of miR-21-5p to endothelial cells increases vascular permeability and angiogenesis.	KRIT1/β-catenin pathway	[71]
Induces Metastasis	Exosomal miR-210 regulates hypoxic environments, enhancing metastatic potential of cancer cells.	HIF-1α pathway	[32]
Facilitates Immune Evasion	Tumor-derived exosomes carry TGF-β1 and IL-10, which increase regulatory T cells (Tregs), reducing immune response against tumors.	SMAD and FoxP3 pathways	[50]
Stimulates Pre-Metastatic Niche Formation	EVs containing lysyl oxidase-like 4 (LOXL4) reprogram stromal cells to support cancer metastasis.	ECM remodeling pathways	[33]
Inhibits Tumor Growth	Engineered exosomes deliver siRNAs targeting oncogenes, reducing tumor growth in xenograft models.	RNA interference pathways	[62]
Enhances Drug Delivery	EVs loaded with chemotherapeutics like paclitaxel show targeted drug delivery with reduced systemic toxicity.	Intracellular trafficking pathways	[64]
Acts as a Biomarker	Elevated miR-3157-3p in exosomes of non-small cell lung cancer patients indicates metastasis.	TIMP/KLF2 pathway	[74]

### 6.3. Exosomes and Cancer

While some exosomal miRNA, such as miRNA25-3p and miR-3157-3p, have been shown to promote tumor metastasis, others have been reported to suppress tumor growth. For instance, a study by Katakowski and colleagues, who studied the effect of MiR-146b-5p on cell migration, reported that MiR-146b-5p suppresses epidermal growth factor receptor in human cancer cells. Their study showed that both continuous and primary cells have significantly lower levels of MiR-146b-5p compared to normal human astrocytes, and that addition of miR-146b-5p decreased cellular growth, migration, and invasion in vitro [76]. Furthermore, injection of exosomal miR-146b into a primary brain tumor in an animal model significantly reduced the growth of the tumor xenograft [77].

MiR-146b-5p was also reported to suppress the inflammation process by down-regulating the expression levels of the pro-inflammatory mediators in lipopolysaccharide stimulated cells [78]. On the contrary, a study by Geraldo and colleagues claimed that MiR-146b-5p plays an oncogenic role in cancer cells. They claimed that MiR-146b-5p is overexpressed in cancer cells and contributes to anticancer drug resistance, and that suppression of miR-146b-5p improved cellular response anti-proliferative signals, and consequently reduced the cellular proliferation rate [79].

The large number and variety of exosomes has not only allowed them to be investigated for their roles as antiproliferative or proliferative particles, but also as targets for anticancer drugs themselves. Monfared et al., 2019, who studied the potential therapeutic value of exosomes packed with a miR-21-sponge construct in a glioblastoma, reported a reduced cellular proliferation and increased apoptosis by using engineered exosomes packed with a miR-21-sponge construct that consequently suppressed miR-21 and upregulated the target genes PDCD4 and RECK in vitro, and significantly reduced tumor volume in vivo [80]. In our recent study by Sergazy et al., 2024, we noticed that mare’s milk-derived exosomes alone were able to reduce a doxorubicin-induced oxidative stress level, albeit not significantly [67]. This observation allowed us to hypothesize that exosomes could be used as an additional therapy to reduce the toxic oxidative stress effects of anticancer drugs such as doxorubicin. Nevertheless, there are many types of exosomes secreted by various cell types, both healthy and diseased, with many unknown functions, and their roles in promoting or suppressing cancer and other pathologies are yet to be determined.

## 7. Exosome Isolation

One of the major challenges in exosomal studies is the suitability of using a reliable exosome isolation methodology. There is no single standard approach, making successful isolation of cell-derived exosomes an emerging research area [81]. Different physical, physicochemical, and immunological techniques for the isolation of cell-derived exosomes are described by the International Society of Extracellular Vesicles (ISEV), which outlines several recommendations for the isolation and characterization of exosomes [82,83]. However, the characterization of single exosomes, such as size, structure, and chemical composition, is very important in selecting the right isolation methodology that will increase the purity and yield of exosomes as well as guiding their evaluation as drug carriers for therapeutic applications [82]. Table 2 shows the most commonly used methods for isolating exosomes.

Differential ultracentrifugation is well suited for the isolation of exosomes, and offers the purest form of exosomes, due to its highly intact biological agents [84]. Ultracentrifugation has also been used to derive exosomes from different fluids, such as milk [85,86]. A number of researchers have suggested the use of ultracentrifugation as the preferred and most readily available method for isolating exosomes [87,88]. Further, Helwa and colleagues found that three distinct exosome isolation kits, namely miRCURY, ExoQuick, and Invitrogen Total Exosome Isolation Reagent, are feasible options for differential ultracentrifugation, especially in cases of limited samples [84].

Recent data indicate that acidification might also be beneficial for the isolation of and efficient release of exosomes; in particular, acidification with hydrochloric acid (HCl) and acetic acid (AA) leads to the formation of ~124 nm and 132 nm exosomes, respectively [42]. The resultant exosome particle concentrations are 2.6 × 10^9^, 1.7 × 10^9^ and 4.8 × 10^8^ particles per mL, using the AA, HCl and ultracentrifugation (UC) techniques, respectively [89]. Additionally, the morphology and protein concentration are quite similar to those of exosomes originating from UC, which produces considerably more exosomes compared to other techniques [89].

In their recent study, Vaswani et al. showed the isolation of extracellular vesicles from a sample of fluid milk (30–200 nm), utilizing a size exclusion chromatography (SEC) column in an ultracentrifuge, as the most suitable isolation method [9]. The researchers examined 12–16 fractions of these bags to deliver more than 1 × 10^8^ particles/mL with a qEV size exclusion column (Izon Science, Oxford, UK), while ultracentrifugation only yielded ~2.2 × 10^8^ particles/mL [90]. Furthermore, sequential centrifugation, in combination with size exclusion chromatographic isolation, showed high concentrations of isolated exosomes [9]. Their research supports the possibility of scalable exosome isolation of significant quantities [91]. In their empirical study, Kaddour and colleagues established specific turbidi-metric liquid chromatography for particle purification, with high size resolution to obtain high-quality exosomes [92].

However, there are a number of emerging new isolation methodologies based on acoustic and electric force mechanisms that aim to improve the yield and purity of isolated exosomes. For example, acoustic fractionation, an acoustic-based isolation method, is a simple isolation technique that does not require sample labelling [PMID: 36714629]. The process involves subjecting the particles to standing acoustic waves through micro-channels, and this was reported to have an isolation rate of more than 80%, with 98% purity [93].

In addition, Zhang et al. described an electric-force-based isolation method that utilizes an insulator-based dielectrophoretic device that captures EVs and then measures their charge-dependent components in the cytosolic and membrane using electrical impedance spectroscopy [94]. The authors claimed that the system at a certain frequency range was sensitive enough to detect EVs from various conditions, and hence their functionalities as well. Interestingly, Tayebi and colleagues have described the application of combining both dielectrophoretic and acoustophoretic forces to isolate and sort EVs [95]. Using the combined methods, these authors were able to isolate and sort various EV subpopulations with more than 80% recovery and 95% purification.

**Table 2:** Commonly used techniques for exosome isolation.

Technique	Purity	Yield	Advantage	Disadvantage	References
Ultracentrifugation	Heterogeneous purity	100–500 mL	High yield	Time consuming	[96]
Density gradient ultracentrifugation	High	10 mL	High purity	Time consuming	[97]
Isoelectric precipitation		Low	10–20 mL	Low cost	[98]
Size exclusion chromatography	High	10–100 mL	High yield of exosomal proteins	Complex method	[99]
ExoQuick precipitation	Low	1 mL	Easy method	High cost	[84]

## 8. Exosome Characterization

The properties of exosomes are specific and unique. Therefore, some specificity indices are required to ascertain whether the extracted elements are exosomes or not, particularly, size and morphology, which are considered as the basic parameters for the characterization of exosomes [100]. In addition, the International Society of Extracellular Vesicles recommends that two protein types are needed to determine if the extracted components are indeed exosomes and whether it is possible to assess the purity of exosomes obtained from a biological fluid by identifying the presence or absence of non-extracellular vesicle protein structural components that are equidistant from the extracellular vesicle [82,101]. Zhang et al. recommends the use of nanoparticle tracking analysis (NTA) to identify exosome morphology, size, and surface protein markers. Characterization procedures primarily fall into two categories, namely exclusion and inclusion methods [101]. Recently, a number of methods for determination and characterization of exosomes have become available, including the method described by Islam et al., which provided a means of measuring extracellular vesicles without pre-processing [102]. These researchers used the NP-TRFIA approach, which utilizes bio-marked antibodies to target four transmembrane proteins and tumor-associated antigens and directly capture extracellular vesicles from urine and cell supernatants [101]. The results of lanthanide-based markers are then compared for characterization. The signal-to-noise ratio of the technique is 2 to 10 times higher than that of the lectin–chelate test. Compared to Western blot and flow cytometry, this method simplifies the separation phase and saves time [101]. Simultaneously, it can be applied in the identification and assessment of tumor-associated proteins on the extracellular vesicle’s surface; hence, it shows potential in the diagnosis and prognosis of various illnesses. Furthermore, particular phospholipids occurring in the lipid bilayer can also be effective controls in the characterization of exosomes’ presence. Thus, Skotland, Sandvig, and Llorente indicate that these may be considered for developing various markers to characterize exosomes in different models [103].

An analysis of the physicochemical features of exosomes, for example their size, density, and shape, is necessary to ascertain their biological interactions and, therefore, the accuracy of characterization [104]. Various methods, such as NTA, Duckworth–Lewis–Stern (DLS), resistive pulse probing (RPS), and flow cytometry, are commonly utilized to characterize exosomes; however, each of these techniques has its own weaknesses, which must be considered in the analysis [105]. For example, NTA can struggle with distinguishing similarly sized particles, DLS may overestimate sizes in polydisperse samples, RPS is prone to clogging and has a limited size range, and flow cytometry may inaccurately detect smaller particles due to high background noise and often requires fluorescent labeling that could alter exosome properties.

Biophysical, molecular, and microfluidic approaches have been widely used to characterize exosomes. In particular, biophysical methods have been applied to determine the size range of exosomes, including optical particle monitoring NTA, which can estimate the size distribution and concentration of exosomes in the range of 10nm to 2 μM [105]. Another methodology used is exosome motion trajectory, which measures particle velocities [105]. This strategy enables the tracking of the Brownian motion of nanoparticles in a liquid suspension on a particle-by-particle foundation [106]. The NTA method is applicable in measuring the exosomal movement by tracking individual particles via image evaluation, with such motion corresponding to the size of the particle [106]. The results of this approach include the particle’s size, size distribution, concentration, and phenotype. One of the main benefits of using NTA is its potential to detect various extracellular vesicles and to measure small particles of up to 30 nm in diameter. It also has an easy setup, quick turnaround time, and short measuring time. Most importantly, after estimation, the samples can be restored to their original form [107].

DLS is an alternative method for measuring the size of exosomes. DLS’s principle of operation is based on a coherent monochromatic laser beam passing through a suspended particle [108]. An example of DLS is tunable resistive pulse sensing (TRPS), which has emerged as a novel approach. According to Anderson, Lane, Korbie, and Trau, the method can measure the size distribution and concentration of exosomes and can be used in the characterization of colloidal particles with a minimum range of 50 nm [109]. Additionally, flow cytometry can be used to examine surface proteins on exosomes. It can also be used to evaluate or ascertain the cellular origin of individual extracellular vesicles [108,109]. However, these methods still lack the potential for application in diagnosis and clinical research [105]. Therefore, there is a need for a reliable and sensitive methodology that is amenable to use in diagnosis and clinical research.

## 9. Potential Therapeutic Use of Exosomes

The diversity of exosomes, their enriched molecular cargo, and their ability in carrying out cell-to-cell communication further supports the list of functions that they play in maintaining health and stimulating disease [110]. However, their exact effects and the roles that they potentially play in health and disease as well biological processes, such as homeostasis, remain unknown [110]. In theory, exosomes carry the signature of their parent cell, thus this will enable early disease detection, or perhaps help in providing personalized therapy based on biomarkers found on/in exosomes. Using exosomes as early patient-specific biomarkers might provide personalized treatment choices and tailored treatment procedures [111]. Conversely, detecting disease-derived exosomes in early disease stages has proven difficult, due to the scarcity of disease-derived exosomes compared to the vast amount of healthy cell-secreted exosomes [112]. While the various biomolecules contained within exosomes can help in identifying their origin, as well as enabling disease diagnosis and treatment, the heterogeneity of exosomes in biological samples complicates the process [82]. Exosome heterogeneity, which is regulated by the differences in the size, origin and molecular cargo of exosomes, represents a challenge in their potential diagnostic and therapeutic uses [113].

**Figure 3:**
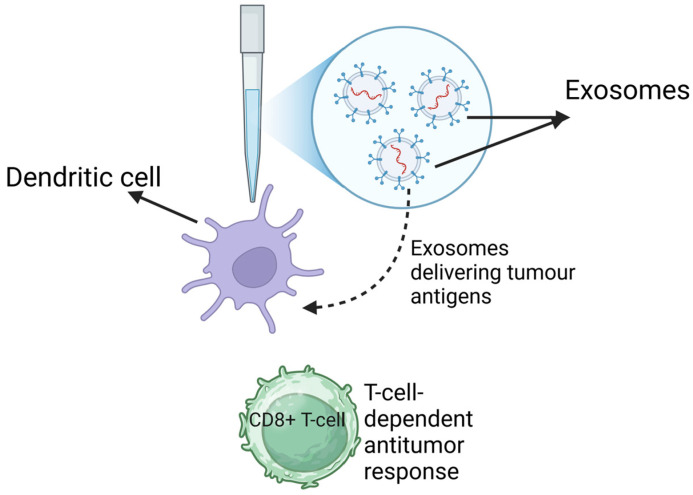
Therapeutic potential of exosomes. Exosomes can be utilized in immune therapies by delivering tumor antigens to dendritic cells, thus initiating a T-cell-dependent antitumor response.

Nevertheless, a number of studies have reported potential therapeutic uses of exosomes and that they present a novel source of therapy, including that of Wolfers and colleagues who, more than two decades ago, claimed the potential use of exosomes as a novel way of delivering immunointerventions [114]. Using a tumor mouse model system, they were able to transfer tumor antigens to dendritic cells using exosomes (Figure 3). After the uptake of the exosomes by the tumor cells, the dendritic cells produced effective CD8+ T-cell-dependent antitumor effects on the mouse tumors [114]. Furthermore, Zitvogel and colleagues claimed that they were able to suppress or eradicate established murine tumors using dendritic cell-derived exosomes [115].

Interestingly, exosomes form a crucial part in the progression and possibly prognosis of breast cancer. For instance, compared to healthy samples, there was significantly higher expression of levels of exosomal–annexin A2 in serum from women with carcinoma, particularly triple-negative breast cancer [116]. In addition, exosomes secreted from pancreatic tumors displayed the membrane bound protein GPC1, which is thought to be a unique biomarker of early disease stage [117]. Guo and colleagues identified an exosome-related signature associated with prognosis and immune infiltration in breast cancer [97]. They used bioinformatics tools to investigate changes in the expression profiles of more than 120 exosomal genes from breast cancer samples, of which seven exosomal-related genes were recognized as a novel prognostic signature for breast cancer [118]. Similarly, the results of using IFN-γ-induced dendritic cell-derived exosomes for the treatment of multiple sclerosis in vivo showed that exosomes were able to transfer a number of miRNAs, specifically miR-219 and miR-9, to the central nervous system (CNS), resulting in myelination in multiple sclerosis as well as demyelination syndromes [119]. Furthermore, a number of clinical trials are currently registered to investigate the potential therapeutic effect of exosomes in various disorders (clinicaltrials.gov). However, the majority appear to investigate their potential as biomarkers and suitability as drug delivery systems. A recent review that analyzed the applications of exosomes in clinical trials reported that the registered clinical trials are mostly investigating exosomes’ use as disease biomarkers, drug delivery systems, cell-free therapeutic agents, and cancer vaccines [120]. This shows the wide scope of clinical applications of exosomes in the treatment and diagnosis of various diseases.

## 10. Discussion

The therapeutic potential and role of exosomes in cancer progression highlight an exciting yet complex avenue for future research and clinical applications. Exosomes, as carriers of bioactive molecules, play a central role in facilitating communication within the tumor microenvironment, aiding in tumor growth, immune evasion, and metastasis. At the same time, their nanoscale size, stability, and capacity to cross biological barriers make them attractive candidates for targeted drug delivery systems.

Tumor-derived exosomes carry a diverse array of oncogenic factors, such as proteins, mRNAs, and microRNAs, which can influence the behavior of nearby cells. These exosomes interact with stromal cells in the tumor microenvironment, reprogramming them into cancer-associated fibroblasts that support tumor progression. By transferring specific signaling molecules, exosomes can also promote angiogenesis, enhance immune evasion, and contribute to the formation of pre-metastatic niches in distant organs. Such functions highlight exosomes as key mediators of cancer progression. Notably, their capacity to facilitate intercellular communication across distances underscores their potential involvement in systemic cancer-related changes, including metastatic spread.

Despite their role in promoting malignancy, exosomes also present a unique opportunity for cancer therapy. Their biocompatibility, low immunogenicity, and ability to encapsulate various therapeutic agents position them as promising drug delivery vehicles. Studies have shown that exosomes can be engineered to carry anti-cancer drugs or genetic material, such as small interfering RNAs (siRNAs) targeting specific oncogenes. This approach could enhance the efficacy of treatments for cancers with limited therapeutic options, such as brain tumors, where exosomes could cross the blood-brain barrier and deliver drugs directly to the site of the tumor, reducing the risk of systemic side effects.

However, realizing the therapeutic potential of exosomes is not without challenges. The heterogeneity in exosome populations—resulting from differences in cell origin, disease state, and environmental conditions—complicates the standardization of isolation and characterization methods. Current techniques, like ultracentrifugation and size-exclusion chromatography, often yield inconsistent results in terms of exosome purity and concentration. This variability limits the reproducibility of studies and poses a significant barrier to clinical translation. In addition, concerns about potential immunogenic responses, variability in exosome cargo and scalability of production methods, present further obstacles.

Future research should focus on refining exosome isolation protocols, developing robust characterization methods to identify disease-specific exosomal signatures, and investigating the influence of exosome heterogeneity on therapeutic outcomes. Advances in bioengineering and molecular biology are likely to provide solutions, enabling the design of exosomes tailored for specific therapeutic applications. Moreover, understanding the mechanisms of cargo loading and targeted delivery could improve the precision and efficacy of exosome-based therapies.

In conclusion, while exosomes hold considerable promise as tools for cancer therapy, their dual role as mediators of tumor progression necessitates careful consideration. Addressing the challenges associated with their clinical application will be crucial in realizing their full potential as vehicles for targeted therapy. Exosome research remains a rapidly evolving field, and as we gain deeper insights into their mechanisms, the likelihood of safe and effective exosome-based treatments becomes increasingly attainable.

## 11. Conclusions

This review provides an overview of the current understanding of exosomes and their potential use in disease diagnosis and therapy. It shows that exosomes have a significantly wide variety of potential uses, ranging from aiding as vehicles for targeted drug delivery to acting as biomarkers for early disease detection and treatment. On the one hand, their nano-scale size, enriched molecular cargo, and the fact that they are secreted by nearly all cells, give them an advantage over other extracellular vesicles. On the other hand, these characteristics present significant challenges for their further development, hindering their future use. Despite the significant progress made in understanding exosomes and the potential roles that they could play in health and disease, there appear to be a lot more questions and challenges to solve, in terms of their suitability in clinical use. Finally, exosomal research is an emerging area that warrants further study.

## Data Availability

No new data were created or analyzed in this study. Data sharing is not applicable to this article.
